# CONTEXTS OF COGNITIVE DECLINE: MAPPING NEIGHBORHOOD OPPORTUNITIES AND BARRIERS FOR HEALTHY AGING IN PLACE

**DOI:** 10.1093/geroni/igac059.553

**Published:** 2022-12-20

**Authors:** Jessica Finlay, Robert Melendez, Philippa Clarke

**Affiliations:** University of Michigan, Ann Arbor, Michigan, United States; Social Environment and Health Program, Institute for Social Research, University of Michigan, Ann Arbor, Michigan, United States; University of Michigan, Ann Arbor, Michigan, United States

## Abstract

Stark geographic variation in later-life health outcomes suggests that local built and social environments are critical in shaping disease and disability, physical and cognitive function, and engagement in everyday life among older adults. This paper presents a community-engaged mapping project aiming to depict the uneven distribution of hazards and amenities relevant to cognitive aging across the United States. Living in neighborhoods with opportunities for social interaction (e.g., coffee shops, senior centers), intellectual stimulation (e.g., museums, libraries) and physical activity (e.g., parks, walkable streets) may slow rates of cognitive decline and reduce risk for Alzheimer’s disease. We assembled a community advisory board to translate research findings into a pilot website and interactive map that assesses neighborhoods for cognitive aging resources and amenities. The objective is to increase public awareness and inform public health and policy efforts to ameliorate community barriers and create more equitable opportunities to promote healthy aging in place.

